# Supplementary material for: Maintenance of Ligament Homeostasis of Spheroid-Colonized Embroidered and Functionalized Scaffolds after 3D Stretch
Source: Int J Mol Sci. 2021 Jul 30;22(15):8204. doi: 10.3390/ijms22158204 (PMC8348491; doi:10.3390/ijms22158204)
Supplement: Supplementary file 1 [file ijms-22-08204-s001.zip › ijms-1284648-supplementary.pdf]

Supplementary Materials:

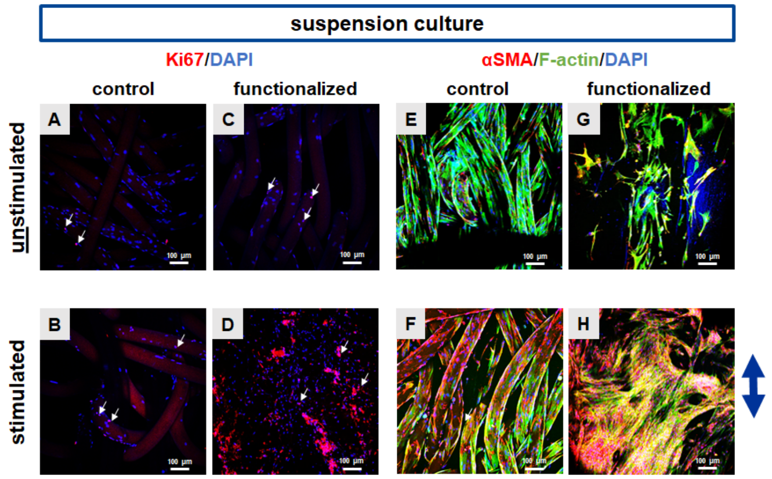

Supplemental Figure S1: Merged images depicting the Ki67 and α-SMA protein expression in scaffolds seeded with the suspension culture. Suspension preculture was performed for 24 hours dynamically. Control (**A, B**) and functionalized scaffolds (**C, D**) were immunocytochemically stained for Ki67. Immunocytochemical staining of the myofibroblast marker α -smooth muscle actin (αSMA, red) combined with F-actin staining (green) was performed for control (**E, F**) and functionalized scaffolds (**G, H**). Unstimulated (**A, C, E, G**) scaffolds were compared with stimulated (72 hours, 4% stretch, 0.11 Hz, **B, D, F, H**). Cell nuclei were counterstained using 4′,6′-diamidino-2-phenylindol (DAPI, blue). The blue arrow on the right side indicates the stretch direction. Scale bars: 100 μm. Three independent experiments with cells of three different donors were performed. Unstim=unstimulated, stim=stimulated.

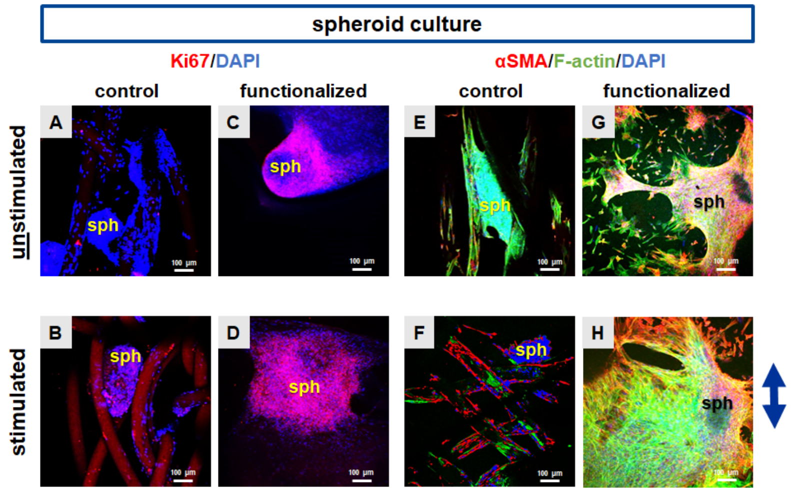

Supplemental Figure S2: Merged images depicting the Ki67 and α-SMA protein expression in scaffolds seeded with spheroid culture. Scaffolds were statically precultured for 5 days. Immunocytochemical staining of Ki67 (red) was performed for control (**A, B**) and functionalized scaffold (**C, D**). Immunocytochemical staining of the myofibroblast marker alpha α-smooth

muscle actin (αSMA, red) combined with staining of F-actin (green) was performed for control (**E, G**) and functionalized scaffolds (**F, H**). Unstimulated (**A, C, E, G**) scaffolds were compared with stimulated (**B, D, F, H**). Cell nuclei were counterstained using 4′,6′-diamidino-2-phenylindol (DAPI, blue). The blue arrow on the right side indicates the stretch direction. Scale bars: 100 μm. Three independent experiments with cells of three different donors were performed. Unstim=unstimulated, stim=stimulated.
